# Calculation of Compound Intake Levels Using Integrated Food Compound Databases and Food Intake Data

**DOI:** 10.1016/j.cdnut.2025.107612

**Published:** 2025-12-10

**Authors:** Marie Y Meima, Joost Westerhout, Sabina Bijlsma, Fiona DM van Schaik, Bas Oldenburg, Marjo JE Campmans-Kuijpers, Marjolein Meijerink, Geert F Houben

**Affiliations:** 1 The Netherlands Organization for Applied Scientific Research, Utrecht, The Netherlands; 2 Department of Gastroenterology and Hepatology, University Medical Center Utrecht, Utrecht, The Netherlands; 3 Department of Gastroenterology and Hepatology, University of Groningen, University Medical Center Groningen, Groningen, The Netherlands; 4 Center for Translational Immunology, University Medical Center Utrecht, Utrecht, The Netherlands

**Keywords:** food intake, food frequency questionnaire, NEVO, FooDB, Phenol-Explorer, food composition databases, food compounds, dietary data

## Abstract

**Background:**

Despite substantial research, the mechanisms through which food influences disease largely remain unclear. Analyzing cohort data at the level of food compounds (i.e. individual molecules present in foods) may reveal new insights, and as a first step, we previously integrated 3 food compound databases.

**Objectives:**

This study aimed to combine the integrated food compound databases with food intake data to estimate compound intake levels and evaluate their plausibility.

**Methods:**

We used food frequency questionnaire (FFQ) records from 135 adult patients with inflammatory bowel disease from University Medical Center Utrecht. Generic FFQ-based dietary data were translated into compound intake values. We dealt with differing compound values across the databases by applying a systematic prioritization strategy to obtain a single representative value per compound in each food item. Intake amounts of 770 compounds were calculated for all 135 subjects. For vitamins and minerals, plausibility was assessed by checking whether the p50 of our calculated intake fell within the p5 to p95 range of Dutch population intake data. For fatty acids and polyphenols, plausibility was assessed by evaluating whether percentile ranges overlapped with those reported in literature.

**Results:**

Our findings indicate that all p50 values of our calculated intake data fell within the p5 to p90 range of the Dutch population intake data for vitamins and minerals. For fatty acids and polyphenols, intake ranges overlapped with those reported in literature for all compounds, but showed more deviation, likely due to regional dietary differences and the absence of standardized population-based intake benchmarks.

**Conclusions:**

Our study provides a foundation for food-health research by offering plausible intake estimates for a wide range of dietary compounds derived from Dutch cohort data.

## Introduction

Decades of research has consistently demonstrated that food significantly impacts health and disease. Diet affects the risk of cancer and many chronic conditions, such as cardiovascular disease, inflammatory bowel disease (IBD), asthma, chronic obstructive pulmonary disease, atopic dermatitis, food allergies, and arthritis [[1], [2], [3], [4], [5], [6], [7]]. Recent findings also indicate that food influences mental well-being [8].

Despite substantial research efforts, the exact mechanisms by which food affects the pathophysiology of diseases remain incompletely understood. One of the major hurdles to unravel the relationship between food and health is the complexity arising from the vast diversity of different molecules present in food, hereafter referred to as “compounds.” Thousands of plant and animal species are consumed as food by humans across different regions of the world, contributing to far >100,000 different compounds identified in these diverse foods [9]. In the past, most research focused on a limited number of these compounds, such as vitamins and minerals, or grouped nutrients (carbohydrates, fats, and proteins) and food categories (dairy, vegetables, and meat). In this way, the health effects of the wide spectrum of individual compounds in food or the effects of specific compound combinations cannot be retrieved. Breaking down food products into individual compounds may ultimately help to clarify how specific dietary components relate to health and disease.

We previously coupled food items from the Dutch food coding system Nederlands Voedingsstoffenbestand (NEVO) to food items within the international food compound databases FooDB and Phenol-Explorer (PE) [10]. This paper extends this work by presenting a detailed methodological framework for combining these databases with food intake data from human cohort studies. This framework was applied in a previous study, in which we conducted statistical analyses on an IBD cohort using both the integrated databases and the combined intake data, aimed at investigating possible differences in compound intakes between patients experiencing flares and those remaining in remission [11]. Although such analysis may be valid for such purpose investigating relative differences in compound intakes between groups, absolute intake estimates may show some differences with actual intakes in the population. In this study, we focus on describing the method itself in more detail and on assessing whether it produces plausible intake estimates. The current framework allows systematic exploration of an expanded set of individual food compounds using dietary data, extending the number of compounds included far beyond the limited set of well-studied nutrients. With this methods paper, we aim to provide a clear and detailed description of our approach, enabling researchers to reproduce and apply it in future compound-centered studies.

## Methods

### Food compounds databases

The food compound databases FooDB (version uploaded on website on 7 April, 2020) and PE (version 3.6, 2016) were previously coupled to NEVO (NEVO online version 2019/6.0, RIVM) [10], which enabled the translation of generic food items into constituent food compounds. NEVO is a Dutch national food compounds database, providing information on 100 individual compounds, including vitamins, minerals, carbohydrates, fats, sterols, polyols, organic acids, and trace elements, present in 2152 foods including raw commodities and processed food items [12]. FooDB is a large international food compounds database that integrates multiple other databases, including Dr. Duke’s Phytochemical and Ethnobotanical Databases, USDA, and Technical University of Denmark, and primarily comprising data on lipids, aromatic compounds, heterocyclic compounds, benzenoids, and organic acids. The downloaded data file of FooDB included 9461 unique food items, with quantitative data for 2634 unique compounds [10]. PE is a dataset including 458 food items with 37,016 concentration values for 508 polyphenols (PE, version 3.6).

In brief, the coupling of these databases involved matching NEVO-coded food items with food items from FooDB and/or PE [10]. The matching was partially automated: an initial rough matching was performed using word similarity via Levenshtein distance. The subsequent selection of the correct matches was carried out entirely manually using 2 different food matching types: “similar matches” referring to food items that were considered identical between NEVO and FooDB or PE, and “processing matches,” referring to matches of food items that underwent distinct processing methods, such as raw food compared with cooked food. The handling of retention factors is described as well in Meima et al. [10]. The matching categories were used to guide data selection for the calculation of compound intakes in the current study ((see section selection and prioritization of compound levels in foods section). As a result of the coupling, the final number of unique compounds available for analysis was 1160.

### Food intake data

The food intake dataset of a cohort of patients with IBD from the University Medical Center Utrecht (UMCU) that we also used for analyzing food compound intake differences between IBD patient groups [11] was reused. The data included food intake and health status information for 135 patients with Crohn’s disease, ulcerative colitis, or IBD unclassified. Flares were defined as *1*) a step-up in IBD medication, *2*) endoscopy showing active disease, *3*) hospitalization due to active IBD, or *4*) surgery related to IBD. Patients were aged between 19 and 68 y (56% female). Further information about the cohort can be found in Opstelten et al. [13]. We used a subset of 94 patients from this cohort, i.e. those who remained in remission for 2 y after the dietary assessment [11]. This group was considered most suitable for food compound intake comparison with the general population.

Dietary assessment was previously done by a food frequency questionnaire (FFQ), capturing the intake of foods consumed during the previous month to assess the individuals’ habitual dietary intake. A specifically adapted questionnaire with additional questions tailored to patients with IBD, including 232 food items, was used [13]. This FFQ was adapted from an earlier 183-item questionnaire designed to capture ≥96% of absolute food intake and ≥95% of between-person variability of nutrients under study, as assessed in the Dutch National Food Consumption Survey (DNFCS) 1998 [14]. Moreover, commonly consumed manufactured food products introduced after 1998 were selected from the DNFCS 2011 and incorporated. In the adapted version, additional questions were included for specific food groups: meat (red, white, and processed), dairy products (fermented and nonfermented), and vegetables (leafy, yellow, and cruciferous). Wageningen University-trained dietitians conducted quality checks for reporting errors and extreme values to safeguard data quality.

The FFQ-collected dietary data, which consisted of aggregated food items such as “leafy vegetables,” were previously processed by Wageningen University, where each FFQ-listed food was broken down into more detailed components and coded according to the Dutch food coding system NEVO. Intake estimates for these NEVO items were weighted based on their consumption proportions in the DNFCS [15]. As such, intake data consisted of the estimated quantities of NEVO items consumed by each subject. These items were converted to compounds data (next section).

### Selection and prioritization of compound levels in foods

**FIGURE 1:**
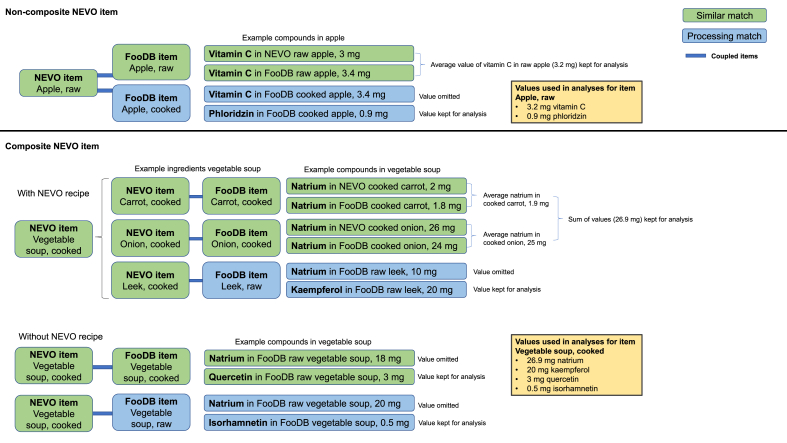
Strategy for the establishment of levels of compounds, using a fictive example of compound levels in the items apple and vegetable soup from the Dutch national food compounds database (NEVO). Note that where only FooDB is mentioned, it may alsrepresent Phenol-Explorer (PE). Levels are displayed in mg/100 g of food item. The strategy is explained in Selection and prioritization of compound levels in foods section. NOVA, Nederlands Voedingsstoffenbestand.

In our previous work [10], we coupled NEVO items to FooDB en PE items, and presented the corresponding compound values per database. The current study builds on that approach by integrating those values into a single, consolidated compound level for each NEVO item. Using compound data from 3 databases involved the handling of differing concentration values of the same compound in a single food item. For instance, the food item “apple, raw” displayed different vitamin C levels in NEVO and FooDB. Also, both a similar match and a processing match could be available for a NEVO item. To obtain 1 unique level for individual compounds in food items, we developed a selection and prioritization strategy, as displayed in Figure 1.

Noncomposite NEVO items were matched one-to-one with entries in FooDB and PE. For example, a raw apple from NEVO was matched with “raw apple” from FooDB and PE (similar match) and with processed types of apple, such as cooked apple (processing match). Compound values from similar matches were prioritized over processing matches in calculating compound intakes (see, for instance, omission of vitamin C in cooked apple in FooDB, given availability of values for raw apple in NEVO and FooDB, Figure 1). Equally derived values were averaged, e.g. if the concentration for vitamin C in raw apple was present in more than one of the databases (NEVO, FooDB, and PE), these vitamin C levels from raw apple were averaged, ignoring vitamin C from cooked apple (Figure 1). If a value for a specific compound was not available in NEVO or from a similar match with FooDB or PE, the values from processing matches were used (e.g. phloridzin in Figure 1), and again averaged in case of multiple values for processing matches.

For composite food items, NEVO Recipes [16] were used to disaggregate food items (e.g. vegetable soup) into their disaggregated counterparts (carrot, celery, and leek) with an original NEVO code, which was based on contribution data of these counterparts derived from the DNFCS. Some of these items were further split within NEVO Recipes into their individual ingredients, each having an original NEVO code (e.g. a “meat ball with egg” was split into meat, bread, and egg); these ingredient data were derived from a general cookbook and occasionally from ingredients on the label [17]. The intake of compounds was calculated based on these disaggregated counterparts and ingredients. On calculating the intake of compounds, we prioritized compound values from the ingredients (e.g. carrot in Figure 1) when a NEVO Recipe was available, rather than those from a similar composite food item from FooDB or PE (e.g. FooDB vegetable soup and cooked). This approach was taken as we considered the Dutch common recipe to better represent the most likely composition. Again, similar matches were prioritized over processing matches, and equally derived values were averaged. Thus, for composite items, the priority order for compounds was: *1*) compounds given for ingredients from NEVO Recipes with a similar match, *2*) compounds given for the composite product with a similar match, *3*) compounds from ingredients with a processing match, and *4*) compounds given for the composite product with a processing match.

### Calculation of compound intake amounts

To standardize the compound concentrations across multiple food composition databases, all values were initially converted to milligrams per 100 g of food item (mg/100 g). Using individual food consumption values (g/d), we then calculated the daily intake of each compound in mg/d for each subject. Thus, the estimated daily intake of individual NEVO items (g/d) was multiplied by the standardized compound concentrations (mg/100 g) to calculate daily compound intakes (mg/d). The calculation of vitamin intakes required additional steps. In brief, unit conversions for vitamin A, vitamin D, vitamin E, and niacin were obtained from the US Food and Drug Administration, 2019 [18]. All compound equivalents for vitamin A, vitamin E, folate, and niacin present in FooDB, expressed as “per kg food item,” were converted to standard equivalents, expressed in mg/100 g. Vitamin D3, expressed as international units, was converted to mg/100 g by multiplying with 0.025 [18]. Hence, all compounds were expressed in similar units and denominators (i.e. mg/100g). More details can be found in [10]. Finally, intake levels of 770 compounds were calculated in mg/d for 94 subjects from the UMCU cohort.

### Comparison of calculated compound intake levels with general population data

We searched literature for appropriate intake data of individual food compounds representative of a Dutch population and compared these with intake levels calculated using our approach to assess whether our estimates are within a plausible range. We used a subset of the IBD cohort data, i.e. the patients that remained in remission during disease follow-up [11], which were 94 patients in total. Intake levels of 9 vitamins and 7 minerals could be compared with those derived from the DNFCS 2007 to 2010 [19], where dietary intake was assessed using 2 nonconsecutive 24-h recalls. Vitamins and minerals have been extensively studied, and their population-based intake estimates are well-established and included in national food composition databases. We considered our calculated intake levels plausible when the p50 fell within the p5 to p95 range of the comparator data (DNFCS 2007–2010). The comparison was not intended as a statistical comparison, because the purpose of this study was to describe our method and assess whether compound intake values derived through our method are in a similar range as those published in literature, rather than to compare dietary intake across cohorts or to assess health effects.

For less-well-studied fatty acids and polyphenols, well-established population intake data are not available. These compounds have received less research attention over the years compared with vitamins and minerals, resulting in limited intake data and their general absence from national food composition databases. Therefore, for fatty acids and polyphenols, we did not apply the criterion used for vitamins and minerals to compare intakes. Instead, we assessed whether the order of magnitude of our intake estimates was comparable to those reported in the literature by checking if there is overlap between the percentile ranges, acknowledging that the available data do not allow for precise benchmarking. Literature on intake levels for 5 fatty acids and 9 polyphenols was identified using perplexity.ai, with the command “provide intake levels for the Dutch population for [compound (group) name]” (February 2024). This search resulted in 2 papers that were deemed useful: McKeever et al. [20] and Gil-Lespinard et al. [21]. Both studies used data from the European Prospective Investigation into Cancer and Nutrition, which includes centers from multiple countries. The fatty acids and polyphenols selected for comparison were based on their presence in both our dataset and the reference datasets. Intake levels of fatty acids were compared with values from a random sample (*n* = 17,000) of the Dutch adult population (20–59 y) who were part of the Monitoring Project on Risk Factors for Chronic Diseases, the European Prospective Investigation into Cancer and Nutrition (MORGEN-EPIC) study [20]. Dietary data in the MORGEN-EPIC study were assessed by a 178-item FFQ. Intake levels for polyphenols were not available for the Dutch population. Therefore, European cohort data from the European Prospective Investigation into Cancer and Nutrition–Physical Activity, Nutrition, Alcohol, Cessation of Smoking, Eating Out of Home and Obesity (EPIC-PANACEA) study were used [21], which included a random sample of 349,165 participants, with the majority (>80%) reported as healthy at baseline. Dietary intake in the EPIC-PANACEA cohort was assessed primarily using semiquantitative FFQs or dietary histories, with some centers additionally using multiple 24-h recalls or food records as reference methods.

Percentiles were plotted to provide a descriptive visualization of intake distributions. We plotted the p5, p50, and p95 percentiles for vitamins, minerals, and polyphenols for both our cohort population and the general population. For fatty acids, we plotted the p20, p50 and p80 as the reference data, only provided these percentiles.

## Results

### Comparison of estimated compound intake levels with general population data

**FIGURE 2:**
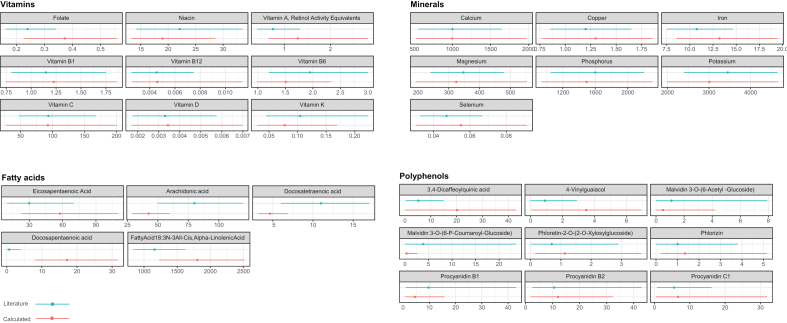
Comparison of calculated intake levels of vitamins, minerals, fatty acids, and polyphenols (red lines) with values from national and European cohort studies (blue lines) expressed in milligrams per day. Dots represent the 50th percentile (median) intake levels; lines indicate the p5 to p95 range (vitamins, minerals, polyphenols) or p20 to p80 range (fatty acids). Comparator values for vitamins and minerals were derived from the DNFCS 2007 to 2010 [18]. Fatty acid intakes were compared with data from the MORGEN-EPIC study [19]; polyphenol intakes were compared with values from the EPIC-PANACEA study [20]. DNFCS, Dutch National Food Consumption Survey; EPIC-PANACEA, European Prospective Investigation into Cancer and Nutrition–Physical Activity, Nutrition, Alcohol, Cessation of Smoking, Eating Out of Home and Obesity; MORGEN-EPIC, Monitoring Project on Risk Factors for Chronic Diseases, the European Prospective Investigation into Cancer and Nutrition.

The 232 FFQ items yielded 894 specific NEVO food items. These NEVO items yielded calculated intake levels for 770 compounds in mg/d for 94 subjects. The p50 values for vitamins and minerals showed general agreement with literature values, with all P50 values falling within the p5 to p90 percentile range of the literature data (Figure 2) [[18], [19], [20]]. The calculated intake levels for fatty acids and polyphenols showed general agreement with literature values, with p20 to p80 or p5 to p95 ranges overlapping in all cases except for docosapentaenoic acid (Figure 2). The method for calculating compound intakes did not show any tendency toward a systematic overestimation or underestimation in comparison with literature values.

Generally, vitamins and minerals showed the strongest similarity with the reference data. Vitamin B1, vitamin B12, vitamin D, niacin, and vitamin C strongly overlapped with those from literature, both in terms of range and median intake (p50). For folate and vitamin A, intake ranges in the general population were narrower, and median values were moderately lower compared with those in our study population. In contrast, vitamin B6 and vitamin K intake levels also showed overlapping distributions, but with moderately higher median values in the literature data. For calcium, copper, magnesium, and phosphorus, intake ranges showed large overlap with those from literature, and median intake levels were also highly similar. For iron, potassium, and selenium, intake distributions similarly overlapped to a large extent, with median values that showed slightly more difference between the datasets.

For 4 out of 5 fatty acids, the intake ranges calculated overlapped with those in the literature and showed the same order of magnitude. Docosapentaenoic acid was the exception, with no overlap observed compared with the data from literature (see Discussion section).

All polyphenols showed overlapping intake ranges. The intakes for malvidin 3-O (6-acetyl glucoside), phloretin 2-O (2-O xylosylglucoside), phlorizin, procyanidin B2, and procyanidin C2 were highly comparable, including the median values. Intake levels for 3,4-dicaffeoylquinic acid and 4-vinylguaiacol were higher in our population compared with literature data, with median values differing by a factor of 0.3. For malvidin 3-O (6-P coumaroyl glucoside) and procyanidin B1, intake was somewhat higher in the data from the literature, including the median values. Overall, considering the overlapping intake ranges, no fundamental differences in polyphenol intakes between those calculated and those found in literature appear to exist.

## Discussion

We developed a method to convert generic food intake data into compound-specific intake data, enabling food compound-centric analyses. This method involves a complex workflow due to the integration of multiple databases, variations in compound nomenclature, and the alignment of food items across databases [10]. Calculating accurate compound intake levels was a key objective of this approach. Our findings indicate that ranges of intake levels calculated for a selection of compounds overlap with the ranges reported in literature, underscoring the credibility of our method despite its complexity. We therefore consider compound intake values derived through our approach suitable for use in future studies on the relationship between food compound intakes and health and disease.

We used data from an IBD cohort that was previously used to study possible differences in compound intakes between patients experiencing flares and those remaining in remission [11]. Although the diets of patients with IBD may deviate from those of healthy subjects [13,[22], [23], [24]], which could challenge the validity of using this cohort in the current study, our aim was to show that our approach provides realistic intake values, at least generally consistent with those of the general population found in literature. We demonstrated that our method does not introduce systematic errors in the estimated intake distributions. Furthermore, we examined several compounds in detail and, as discussed below, hypothesize that the differences between results calculated for our cohort and literature data may be primarily due to methodological variations (dietary assessment methods) and local dietary habits.

Vitamin and mineral intakes calculated for UMCU cohort participants showed comparable intake ranges as those found in the DNFCS, with calculated p50 values falling within the p5 to p95 range of the comparator study. A limitation in interpreting the intake estimates for certain fatty acids and polyphenols is that no well-established, population-based reference values are currently available. These compounds have been studied less extensively than vitamins and minerals, and as such, both our estimates and those from comparator studies carry a degree of uncertainty. Because neither dataset can be considered a definitive benchmark, we focused on whether the intake distributions showed overlapping ranges. This approach did not aim to validate our estimates, but rather to explore whether they fell within a comparable order of magnitude. Taking this into account, fatty acid intake levels generally fell within similar ranges compared with Dutch population-based intake levels [20]. The intake of docosapentaenoic acid in our population (p20–p80 range: 8.0–31.7 mg/d) was the only fatty acid that did not overlap with the p20–p80 range reported for the Dutch general population data (p20–p80 range: 0.1–4 mg/d). The median intakes of docosapentaenoic acid were 17 mg/d in our population compared with 0.7 mg/d reported for the Dutch general population. However, other literature indicates higher levels in other European countries, with median daily intakes of 12 mg/d in Belgium and 80 mg/d in Denmark [25]. These differences may reflect variations in dietary habits; for example, docosapentaenoic acid is mainly derived from fatty fish, which is consumed more frequently in the Danish population than in the Dutch general population [26]. These findings reveal variability in reported intake levels for docosapentaenoic among different studies, populations, and/or regions and indicate that the level found in our analysis is well within ranges observed in European populations.

Polyphenol intake levels generally fell within similar ranges compared with intake levels from reference data. Also here, the variability likely reflects the limited coverage of polyphenols in food composition databases. However, we further evaluated the differences found for polyphenols. We found that differences in dietary habits between countries are a likely factor for the relatively larger differences in polyphenol intake values calculated for our population and those found in literature. For example, the polyphenols 3,4-dicaffeoylquinic acid and 4-vinylguaiacol showed higher intake values in our cohort compared with the European cohort data. The intake of these polyphenols was primarily through coffee consumption. In Europe, the Netherlands ranks among the highest in mean daily coffee consumption [27]. The median intake in our study, calculated to be 3 to 4 cups of coffee per day based on phenol intake data, in comparison to ∼2 cups of coffee per day in Knaze et al. [28], aligns with these findings. Research also indicates that patients with IBD tend to consume slightly more coffee compared with the general population [13], potentially adding to the higher levels of intake of polyphenols primarily found in coffee in our cohort. In contrast, malvidin 3-O-(6-P-coumaroyl-glucoside), malvidin 3-O-(6-acetyl-glucoside), procyanidin B1, and procyanidin B2 were found to have lower intake values in our cohort compared with European reference data, likely reflecting lower consumption of red wine, one of the richest dietary sources for these polyphenols. Indeed, red wine consumption in the Netherlands is lower than that in many of the European countries included in the EPIC cohort [29]. Additionally, lower intake of polyphenols from red wine among patients with IBD may be attributed to their reduced consumption of alcoholic beverages [13].

For the other polyphenols, comparing their intake on food source level was not possible due to their presence in a wide variety of products not detailed in Opstelten et al. [13]. However, it is plausible that similar differences between countries, and to a certain extent between patients with IBD and healthy individuals, may apply to other polyphenols, and other compounds, as well. Opstelten et al. [13] have shown that Dutch adults with IBD report higher intakes of animal protein and carbohydrates, and lower intakes of unsaturated fat, dietary fiber, and alcohol compared with controls. Such differences suggest that the dietary intake of patients with IBD may not fully reflect that of the general population, which is important to consider when interpreting the generalizability of our findings. Methodological aspects may also have played some role in differences in intakes calculated for our population compared with literature data: 2 of the comparator studies assessed diet using 24-h recalls, whereas our data were collected with an FFQ. FFQs capture usual intake over a longer period of time by grouping foods and assigning standard proportions of portion sizes, which may not reflect the exact items or amounts actually consumed. For instance, spinach and endive are categorized together as “leafy vegetables” within the FFQ, despite their distinct molecular compositions. In contrast, 24-h recalls record the specific foods eaten on a given day, capturing actual compound content. As a result, FFQs may underestimate or overestimate intake of certain compounds depending on the assumptions made for food group composition, whereas 24-h recalls may show higher or lower intakes due to day-to-day variation or consumption of uncommon, nutrient-rich foods. However, the evaluation of differences for compounds for which such evaluation was feasible (see examples above) indicates that dietary differences between countries mainly explain the differences observed. Dietary differences between countries, and to a certain extent between patients with IBD and healthy individuals, are therefore the likely main factor for the differences found, thus likely reflecting actual existing differences.

Dietary data in our study were collected by FFQs, which capture food consumption based on a predefined food list. Usually, the data reflect food intake from the past month and is intended to represent regular eating habits of subjects. The cohort data we used were based on a 1-time FFQ. As noted above, a notable drawback of FFQs, particularly in the context of a compound-centric approach, is the grouping of foods. Such grouping of foods leads to less reliable compound intake estimates. To mitigate this limitation, food groups were disaggregated into specific ingredients using NEVO Recipes [16]. These recipes were derived from the DNFCS and employed with the assumption that these provided reasonably accurate estimates of actual detailed food intake in the Netherlands [17]. In this way, uncertainties in intake assumptions caused by the FFQ were reduced as much as possible. When using an FFQ for compound-centered analyses, it is essential that the questionnaire is comprehensive and includes all relevant food groups. We consider the cohort data of UMCU suitable for compound-centric analysis, as it captured all major food categories. One limitation of the UMCU FFQ is that it did not capture herbs and spices, which can contain bioactive compounds with potential health benefits. Future food compound-centered studies may benefit from more detailed dietary data collection methods, such as a combined 24-h dietary recall and FFQ.

A major challenge in food and nutritional studies is the scarcity, unreliability, inconsistency, and outdated nature of data in food composition databases [30]. The large majority of the estimated 10,000 of compounds in the human diet remain unidentified and unquantified in current databases. FooDB, although extensive in terms of the types of compounds covered, lacks quantitative data for many compounds. Also, FooDB contains different nomenclature for similar food compounds. Despite multiple automated and manual efforts to identify synonyms and using a single term per compound [10], we still identified compounds for which synonyms were used in our database. Therefore, for food compounds deemed significant in a study, it is recommended to perform an additional manual check for remaining synonyms. Additionally, for vitamin A, folate, and niacin, amounts were sometimes listed separately for various vitamers within the same product, sometimes expressed by various units, which could potentially lead to multiple records for the vitamin. We standardized the compound names and units and added them up across different products to estimate total intakes. However, this approach may occasionally have resulted in adding up different forms of the same compound. Despite our efforts to standardize FooDB nomenclature and units, further improvement of synonym harmonization of food compound databases would be of value.

We presented a method for translating food consumption data into compound intake values. Calculated intakes for vitamins, minerals, and fatty acids were in line with general population data from the same country found in literature, or with data from comparable countries. Polyphenol intakes were also in the same range as those found in literature, though showed relatively more variability between countries, likely mainly due to dietary differences, thereby reflecting actual existing differences. Despite data gaps, application of this compound-centric approach to health and disease cohort research data can improve our understanding of food-health interactions considerably and ultimately pave the way for the use of diet or new food research-derived concepts in (adjunctive) therapy and prevention of diseases. Absolute intake estimates should always be interpreted cautiously, but comparison of relative intakes might reveal meaningful differences between subgroups within a cohort, provided dietary assessment methods capture all significant foods.

## Author contributions

The authors’ responsibilities were as follows – MYM, GFH: designed research and wrote the paper; MYM, JW, SB, MM, GFH: conducted research; FDMvS, BO, MJEC-K: provided data necessary for the research; MYM, JW, SB: analyzed data or performed statistical analysis; JW, SB, FDMvS, BO, MJEC-K, MM: edited the paper; and all authors: read and approved the final manuscript.

## Data availability

Data described in the manuscript will not be made available because it includes clinical information owned by hospitals, which is not allowed to be distributed. Additionally, the integrated food compounds databases belong to TNO and are also not available for sharing.

## Declaration of generative AI and AI-assisted technologies in the writing process

During the preparation of this work, the authors used CoPilot and ChatGPT to improve readability. Additionally, perplexity.ai was used to search for relevant literature on intake levels of food compounds in the general population. After using these tools, the authors reviewed and edited the content as needed and take full responsibility for the content of the published article.

## Funding

This work was supported by the Dutch Governmental TNO Research Cooperation Funds.

## Conflict of interest

FDMvS has received consultancy fees from Takeda and Galapagos, speaker’s honoraria from Galapagos, Lilly, and Janssen-Cilag B.V., hospitality fees from Ferring and Dr. Falk Farma, and an unrestricted research grant from Takeda. BO has received research support from Galapagos, Abbvie, Takeda, Ferring, and consulting and speaker fees from Galapagos, Takeda, Janssen, BMS, Pfzer, Abbvie, and Ferring. The other authors declare no conflicts of interest.
